# The language profile of behavioral variant frontotemporal dementia

**DOI:** 10.3233/JAD-150806

**Published:** 2015-12-10

**Authors:** Chris J. D. Hardy, Aisling H. Buckley, Laura E. Downey, Manja Lehmann, Vitor C. Zimmerer, Rosemary A. Varley, Sebastian J. Crutch, Jonathan D. Rohrer, Elizabeth K Warrington, Jason D. Warren

**Affiliations:** a Dementia Research Centre, Department of Neurodegenerative Disease, UCL Institute of Neurology, University College London, London, United Kingdom; b Dept of Language & Communication, Division of Psychology & Language Sciences, University College London, London, United Kingdom

**Keywords:** behavioral variant frontotemporal dementia, primary progressive aphasia, frontotemporal dementia

## Abstract

**Background:**

The language profile of behavioral variant frontotemporal dementia (bvFTD) remains to be fully defined.

**Objective:**

We aimed to quantify the extent of language deficits in this patient group.

**Methods:**

We assessed a cohort of patients with bvFTD (n=24) in relation to patents with semantic variant primary progressive aphasia (svPPA; n=14), nonfluent variant primary progressive aphasia (nfvPPA; n=18) and healthy age-matched individuals (n=24) cross-sectionally and longitudinally using a comprehensive battery of language and general neuropsychological tests. Neuroanatomical associations of language performance were assessed using voxel-based morphometry of patients’ brain magnetic resonance images.

**Results:**

Relative to healthy controls, and after accounting for nonverbal executive performance, patients with bvFTD showed deficits of noun and verb naming and single word comprehension, diminished spontaneous propositional speech and deterioration in naming performance over time. Within the bvFTD group, patients with *MAPT* mutations had more severe impairments of noun naming and single word comprehension than patients with *C9orf72* mutations. Overall the bvFTD group had less severe language deficits than patients with PPA, but showed a language profile that was qualitatively similar to svPPA. Neuroanatomical correlates of naming and word comprehension performance in bvFTD were identified predominantly in inferior frontal and antero-inferior temporal cortices within the dominant hemispheric language network.

**Conclusions:**

bvFTD is associated with a language profile including verbal semantic impairment that warrants further evaluation as a novel biomarker.

## INTRODUCTION

Among the phenotypes of frontotemporal lobar degeneration, the behavioral variant of frontotemporal dementia (bvFTD) is the most clinically, anatomically and pathologically diverse.[1-5] While patients typically present with social disintegration and personality change,[5,6] there is substantial phenotypic overlap with other entities, in particular the primary progressive aphasias (PPA), even at relatively early stages.[1,5]. Previous studies including data on language functions in bvFTD[6-22] are summarized in Table 1. Deficits in confrontation naming[7,8,9,11,19,22], comprehension of single words[7,18] and sentences[6,10,16] and more generalized semantic and language impairment[12,14,15,17,20] have been described in bvFTD. Regional frontotemporal atrophy in bvFTD often overlaps brain networks canonically concerned with language[2,3] and available neuroanatomical evidence has implicated distributed frontal, temporal and parietal circuitry in the genesis of language deficits in this syndrome[6,8,9,19]. Taken together, this evidence suggests that bvFTD may lead to language dysfunction particularly where there is a requirement for processing verbal associations, searching the verbal lexicon or planning propositional utterances. However, direct head-to-head comparisons between bvFTD and primary progressive aphasia syndromes with simultaneous, comprehensive language assessment and neuroanatomical correlation have been undertaken only infrequently[8,9] (see Table 1). Language deficits in bvFTD remain incompletely defined and may go unrecognized.

Here we addressed this issue with a comprehensive assessment of language and general neuropsychological functions in a well-characterized cohort of patients with bvFTD. The language profile of bvFTD was determined after taking into account general (nonverbal) executive performance, an important potentially confounding factor in this group of patients. The bvFTD cohort was compared to patient cohorts with canonical syndromic variants of primary progressive aphasia, assessed both cross-sectionally and longitudinally, in order to examine the relative salience and chart the progression of any language deficits. Neuroanatomical associations of language impairments were assessed using voxel-based morphometry of patients’ brain magnetic resonance images. Building on the cumulative evidence of previous work, we hypothesized that patients with bvFTD have deficits particularly affecting language functions such as word retrieval and propositional speech that are likely to engage executive processes. We further hypothesized that these deficits particularly implicate anterior subregions of the distributed dominant hemisphere language network.

## MATERIALS AND METHODS

### Participants

Twenty-four patients with behavioral variant frontotemporal dementia (bvFTD), 14 patients with semantic variant primary progressive aphasia (svPPA) and 18 patients with nonfluent variant primary progressive aphasia (nfvPPA) were recruited via a tertiary cognitive disorders clinic as part of a cross-sectional and longitudinal cognitive and neuroimaging study of frontotemporal lobar degeneration. All patients fulfilled current consensus criteria for a probable or definite syndromic diagnosis[1,23] corroborated by general neuropsychological assessment. Genetic screening of the patient cohort revealed pathogenic mutations in 11 cases (five *C9orf72*, four bvFTD, one nfvPPA; six *MAPT*, all bvFTD). Volumetric brain MRI showed compatible profiles of regional brain atrophy in each of the syndromic groups; none of the patients had a significant intercurrent burden of cerebrovascular disease. Twenty-four healthy individuals with no history of neurological or psychiatric illness age-matched to the patient cohort also participated. Demographic, clinical and background neuropsychological data for all participant groups are summarized in Table 2.

All participants gave informed consent, and ethical approval for the study was granted by the National Hospital for Neurology and Neurosurgery and the University College London Hospital Research Ethics Committees, in line with Declaration of Helsinki guidelines.

### Assessment of language

Language tests administered to participants covered seven core domains of language processing: speech input (Psycholinguistic Assessment of Language Processing in Aphasia subtest 3, PALPA3 minimal pairs discrimination),[24] speech repetition (word and sentence repetition),[25] single word comprehension (word – picture matching using the British Picture Vocabulary Scale;[26] concrete and abstract words from the synonyms comprehension test),[27] sentence comprehension (PALPA55 picture-sentence matching task),[24] lexical retrieval (noun naming, Graded Naming Test;[28] a novel verb naming test using pictured actions, further details in Supplementary Materials on-line), reading (Graded Nonword Reading test,[29] National Adult Reading Test;[30] Schonell Graded Word Reading Test)[31] and spelling (Graded Difficulty Spelling test)[32] (see Table 3). Further details of the test procedures are provided in Supplementary Material on-line and the language battery used here has been described previously [33]. In addition, participants’ spontaneous propositional speech was assessed by asking them to describe their last holiday; participants were encouraged to talk for up to three minutes, using prompts if necessary. This procedure was designed to be as open-ended as possible, in order to compass the anticipated very wide range of fluency and general language competence across participant groups; prompts were intended to ensure that the language sample obtained for each participant was as complete as possible (examples of prompts included, “Where did you go?”, “How long were you there for?”, “How did you get there?”, and “What did you do there?”). Both the participants’ responses and any examiner prompts were recorded for offline analysis.

Longitudinal neuropsychological assessments between one and three years apart were conducted for a subset of the bvFTD (n=16; mean (standard deviation) test interval 570 (212) days), svPPA (n=9; 518 (236) days), nfvPPA (n=10; 409 (110) days) and healthy control (n=15; 471 (190) days) groups. The mean test interval did not differ significantly between participant groups.

### Analysis of behavioral data

Propositional speech recordings were first processed to extract for each participant the total number of words, number of words normalized for number of prompts required from the examiner, and median word frequency (British National Corpus, spoken portion, http://www.natcorp.ox.ac.uk/). The proportions of nouns and verbs produced by each participant were calculated by dividing the number of words in each category by total number of words produced, in order to control for overall utterance length.

All behavioral data were analysed using Stata^®^ v12. Demographic characteristics and general neuropsychological data were compared between groups using independent samples t-tests for continuous variables and chi square tests for dichotomous variables. Language performance was compared between groups separately for each graded difficulty language test and for propositional speech variables using a linear regression model incorporating test score as the variable of interest with covariates of gender and WASI Matrices score (an index of general nonverbal executive function and surrogate of disease severity). In order to take account of near-ceiling performance by healthy controls, pass/ fail variables were compared between groups separately using chi-square tests. Post hoc subgroup analyses were conducted to compare bvFTD patients with genetic mutations with other bvFTD cases using independent samples t-tests for continuous variables and chi square tests for dichotomous variables. Neuropsychological performance profiles were constructed for each patient group by transforming raw group mean scores on each test to a z-score relative to the healthy older control group mean where feasible.

Longitudinal language data were analysed using Wilcoxon matched-pairs signed-ranks tests. For all behavioral comparisons, a threshold of p<0.05 was accepted as the criterion for statistical significance.

### Brain image acquisition and analysis

Volumetric brain MR images were acquired for all patients in a 3.0 T Siemen’s Trio MRI scanner using a 32-channel phased array head-coil and a T_1_-weighted sagittal 3D magnetization rapid gradient echo sequence (TE = 2.9msec, TR = 900msec, TI = 2200msec), with dimensions of 256 × 256 × 208, and voxel size of 1.1 × 1.1 × 1.1 mm^3^.

In order to conduct a voxel-based morphometry (VBM) analysis of patients’ neuroanatomical data, brain images were first pre-processed and normalized to NMI space using SPM12 software (http://www.fil.ion.ucl.ac.uk/spm/software/spm12/) and the DARTEL toolbox with default settings for all parameters[34,35] running under Matlab^®^ R2012a. Images were smoothed using a 6mm full-width at half-maximum (FWHM) Gaussian kernel. To control for individual differences in head size, total intracranial volume (TIV) was calculated for each participant by summing grey matter, white matter and cerebrospinal fluid volumes following segmentation. A study-specific template brain image was created by warping all native space whole-brain images to the final DARTEL template and calculating the average of these images.

Voxel intensity (an index of brain volume) was modelled separately in each patient group as a function of language performance for each task on which bvFTD patients showed a deficit in the behavioral analysis. Age, gender, TIV and WASI Matrices score were incorporated as covariates of no interest in all models. An explicit brain mask was applied, whereby a voxel was included in the analysis if grey matter intensity at that voxel was >0.1 in >70% of the participants.[36] Statistical parametric maps (SPMs) were assessed at two significance criteria: thresholded at p<0.05 after family-wise error (FWE) correction for multiple voxel-wise comparisons over the whole brain; and thresholded at p<0.05_FWE_ after small volume correction within subregions of the left hemisphere language network pre-specified in our prior anatomical hypotheses. These anatomical regions comprised inferior frontal gyrus, posterior superior temporal cortex and anterior temporal lobe, derived from the Juelich Histological and Oxford/Harvard defined brain regions in FSL v3.12[37,38] and edited in MRICron^®^ to conform to our customized group template image.[39]

## RESULTS

### Behavioral data

Results of group comparisons for background neuropsychological tasks are presented in Table 2 and language tasks in Table 3. Individual raw performance data are presented in Supplementary Figure S1 on-line.

Participant groups did not differ significantly in age, handedness, or educational attainment and patient groups did not differ in symptom duration. Males were over-represented in the bvFTD group relative to each of the other groups and gender was accordingly incorporated as a covariate of no interest in analyses of language variables. Patient groups showed widespread general (extra-linguistic) neuropsychological deficits and the svPPA and nfvPPA groups showed, as anticipated, specific syndromic language profiles relative to the healthy control group. The bvFTD group showed significantly worse recognition memory for words and significantly better verbal and nonverbal working memory and arithmetic performance than the nfvPPA group; and significantly worse verbal working memory performance than the svPPA group.

**Figure 1:**
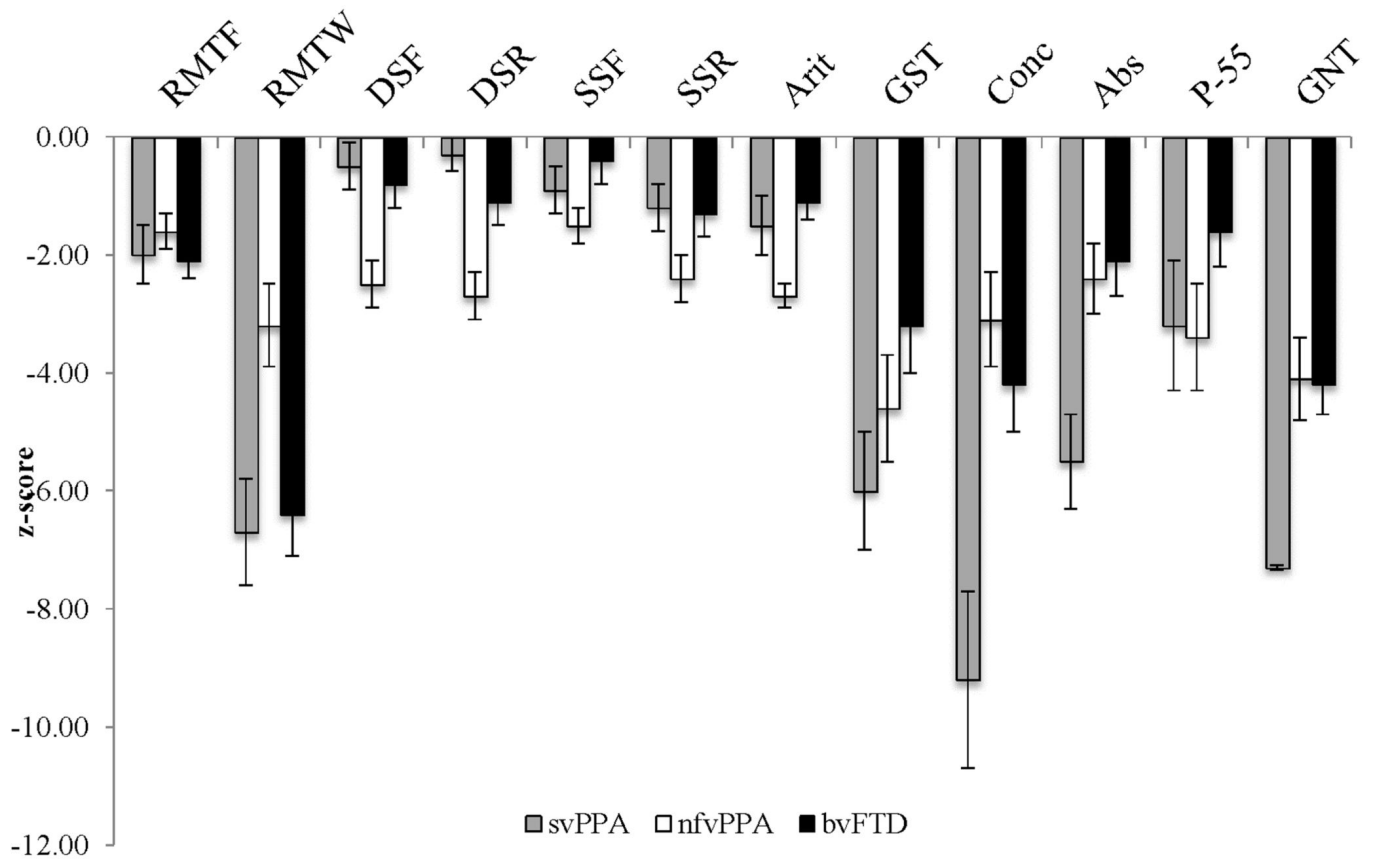
Neuropsychological profiles of patient groups relative to the present healthy older control group. The profiles incorporate tests for which raw scores have been transformed so that 0 represents the mean score of the healthy control group; larger deviations from this thus indicate increasing impairment relative to controls. Error bars represent ± 1 standard error of the mean. Abs, abstract synonyms; Arit, arithmetic; bvFTD, behavioral variant frontotemporal dementia; Conc, concrete synonyms; DSF/R, Digit Span Forward/ Reverse; GDA, Graded Difficulty Arithmetic test; GNT, Graded Naming Test; GST, Graded Difficulty Spelling test; nfvPPA, nonfluent variant primary progressive aphasia; P-55, Psycholinguistic Assessment of Language Performance in Aphasia; RMTF/W, Recognition Memory Test for faces/words; SSF/R, Spatial Span Forward/ Reverse; svPPA, semantic variant primary progressive aphasia. See text for details.

Relative to the healthy control group, the bvFTD group overall showed impairments of noun naming, verb naming and concrete single word comprehension (concrete synonyms). Patients with bvFTD did not show deficits of abstract single word comprehension, sentence comprehension (whether considered overall or separately by PALPA55 grammatical construction categories) or any other language domains. The bvFTD group performed significantly better than the svPPA group on tests of noun and verb naming, single word comprehension (concrete and abstract synonyms), sentence comprehension and spelling; and significantly better than the nfvPPA group on tests of nonword repetition and reading. Performance profiles across tests (based on transformed z-scores for each of the patient groups relative to the healthy control group) are presented in Figure 1.

A more detailed analysis of the bvFTD group (summarized in Table S1 in Supplementary Material on-line) revealed two subgroups stratified by performance on language tasks: a more severe subgroup of 10 patients performing >2 standard deviations below the healthy control group mean on tests of both noun naming and single word comprehension and a less severe subgroup comprising the remaining 14 bvFTD cases. The more severe subgroup was significantly older, had significantly lower MMSE scores and significantly shorter symptom duration than the less severe subgroup. However, there were no consistent profiles of regional brain atrophy on MRI for either subgroup: both subgroups represented a variety of atrophy profiles and in particular, each subgroup contained only a single patient with focal, asymmetric temporal lobe atrophy (see Table S1).

Post hoc analyses of the genetic subgroups within the bvFTD group (summarized in Table 4) revealed that the *MAPT* mutation subgroup performed significantly worse than the *C9orf72* mutation subgroup on noun naming and single word comprehension (British Picture Vocabulary Scale). The *C9orf72* mutation subgroup performed significantly worse than the *MAPT* mutation subgroup on working memory measures (Table 4); no other significant neuropsychological differences between the genetic subgroups were identified.

In the propositional speech analysis, the bvFTD group did not differ from healthy controls in total number or mean frequency of words produced but produced significantly fewer words on average between prompts than the healthy control group. Median word frequency score in the bvFTD group was significantly lower than in both the svPPA and nfvPPA groups. There were no significant differences between groups in the proportions of nouns and verbs produced.

Results of the longitudinal analysis of language data in the participant groups are summarized in Table 5. The bvFTD group showed significant deterioration in noun naming between time-points, while the nfvPPA group showed a significant interval decline in sentence comprehension and the svPPA group showed a significant decline in single word comprehension. The subgroup of bvFTD patients less severely affected at baseline showed a longitudinal decline in naming (Table S1), indicating this effect was not restricted to more severely affected patients with bvFTD.

### Voxel-based morphometry data

**Figure 2:**
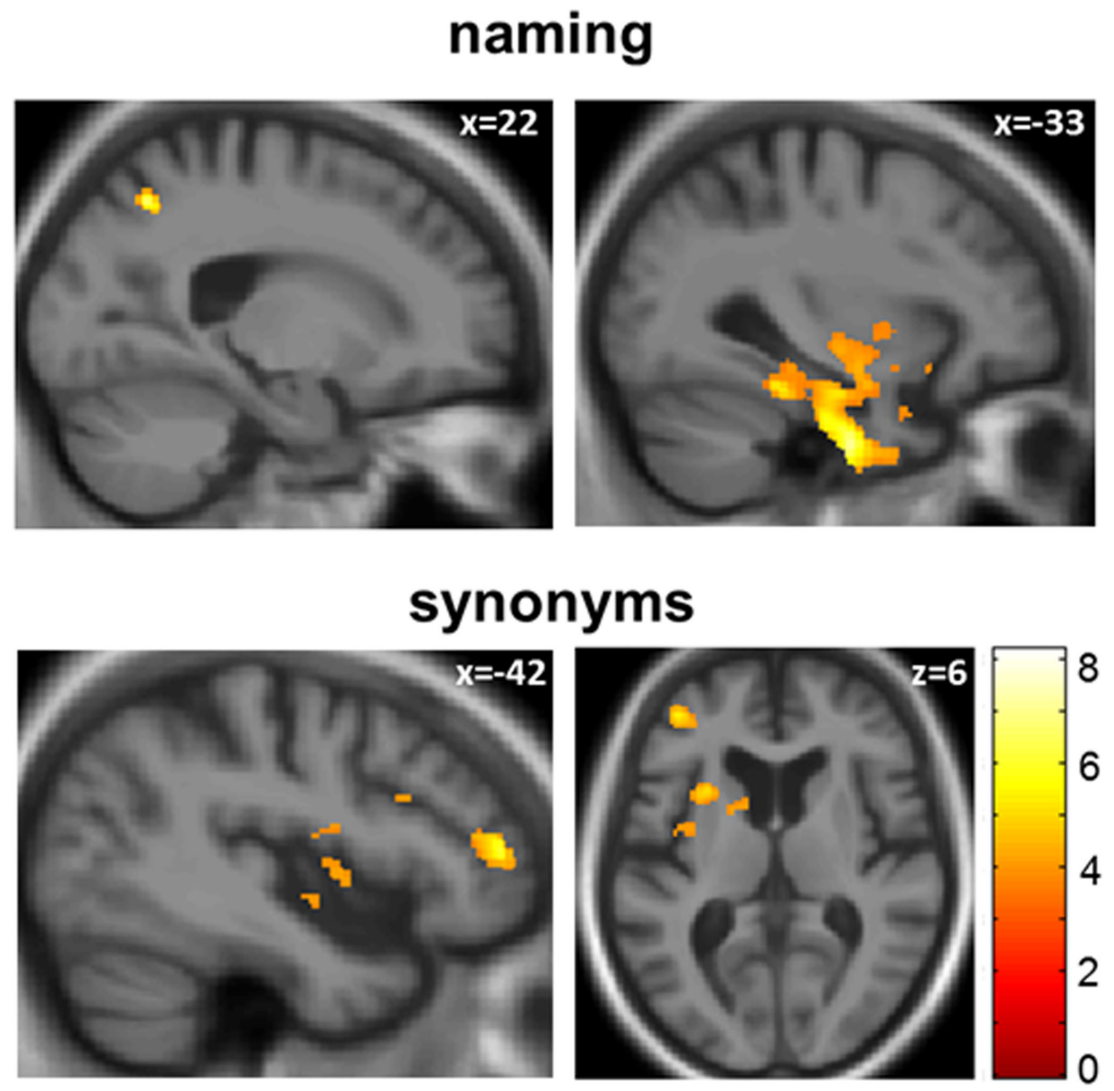
Statistical parametric maps (SPMs) of regional grey matter loss associated with impaired performance on tests of word retrieval (Graded Naming Test, above) and single word comprehension (concrete synonyms test, below) in the patient group with behavioral variant frontotemporal dementia are shown. SPMs are thresholded at p < 0.001 uncorrected for display purposes (all associations significant at p<0.05_FWE_ for multiple corrections over the whole brain or within the prespecified anatomical region of interest, see Table 2), and displayed on sections of the mean normalized T1-weighted structural brain MR image; the left hemisphere is presented on the left in the axial section and MNI coordinates for the plane of each section are indicated. The color bar codes the range of Z-scores for each SPM.

Neuroanatomical associations with language deficits in the bvFTD group (noun naming and concrete single word comprehension) are compared with the svPPA and nfvPPA groups in Table 6; statistical parametric maps of associated regional grey matter atrophy in the bvFTD group are presented in Figure 2.

In the bvFTD group, worse noun naming performance was associated with decreased grey matter volume in right superior parietal cortex and left anterior fusiform gyrus (p<0.05_FWE_ for multiple voxel-wise comparisons over the whole brain), the cluster extending toward the left temporal pole. Worse noun naming performance in the nfvPPA group was associated with decreased grey matter in left middle temporal gyrus/superior temporal sulcus (p<0.05_FWE_ within pre-specified anatomical region of interest); no significant associations between noun naming and grey matter volume were identified in the svPPA group. In the bvFTD group, worse single word comprehension performance was associated with decreased grey matter volume in left inferior frontal gyrus and inferior frontal sulcus (p<0.05_FWE_ within pre-specified anatomical region of interest, see Figure 2 – though note that SPMs are presented at p<0.0001 uncorrected for display purposes); no significant associations of single word comprehension were identified in the svPPA or nfvPPA groups. No significant grey matter associations of reduced propositional speech output were identified. Reverse contrasts of each language variable did not yield significant grey matter associations in any group.

## DISCUSSION

Here we have shown that language deficits accompany bvFTD even after taking general executive performance and disease severity factors into account. These deficits were particularly prominent in the domains of single word comprehension (as indexed using the synonyms test) and lexical retrieval (as indexed using the Graded Naming Test). While naming is a multi-component cognitive process, the naming deficit demonstrated in the bvFTD group here may be at least partly semantically based. The present findings corroborate previous evidence for impairments of naming and verbal semantic functions in bvFTD[7,8,9,11,18,19,22] and suggest that a more specific, primary verbal semantic deficit may be a core linguistic feature of the bvFTD syndrome. In line with this, a profile analysis (Figure 1) revealed a qualitatively similar pattern of deficits in the bvFTD and svPPA groups here. While the patient groups were selected according to current consensus criteria for the respective syndromes, this resonates with clinical experience and previous neuropsychological work suggesting convergence in the behavioral and cognitive profiles of bvFTD and svPPA[33,40,41]. It is unlikely the findings are attributable simply to misclassification of svPPA cases since the most lexically impaired patients in the bvFTD cohort considered as a subgroup did not show a profile of regional brain atrophy compatible with svPPA. It is of interest that decline in naming performance over time was a signal of disease evolution in the bvFTD group but not in the PPA groups here. While this apparent discrepancy may be at least partly attributable to the relatively small size of the present PPA groups and floor effects in the svPPA group, our data suggest that naming as a general index of language function may be a candidate biomarker in bvFTD. This is pertinent given the current paucity of biomarkers in bvFTD and the difficulties surrounding measurement of the complex social and emotional behaviors that typically dominate the clinical picture in this syndrome.

Neuroanatomical correlates of naming and word comprehension in bvFTD were identified in a distributed, predominantly left-lateralized and anterior network of cortical regions, including anterior and inferior temporal and inferior frontal cortices. These areas are canonical components of the language network and have been previously implicated in word retrieval and control processes both in the healthy brain and in lesion studies.[42,43] Similar prefrontal cortical correlates of naming performance have been identified in previous work in bvFTD.[9] While the nondominant parietal correlate of naming performance shown here appears at first more surprising, strength of activation in this region during a semantic decision task has been correlated with off-line naming performance in the healthy brain[44] and its engagement here may reflect cross-modal integrative mechanisms during the picture naming task or semantic task load in these cognitively impaired individuals.[42] It is noteworthy that the bvFTD group also had reduced spontaneous generation of propositional language. While a direct neuroanatomical correlate of propositional speech output was not identified here, this is likely to be grounded in a similar anterior dominant hemispheric network, based on evidence in the healthy brain.[45]

Differential involvement of these networks may also account for the stratification of language profiles between the *MAPT* and *C9orf72* genetic mutation subgroups in this study. Though case numbers were not sufficient for direct neuroanatomical correlation, these mutation subgroups have been shown previously to have distinct neuroanatomical profiles, with relatively focal involvement of anterior temporal and inferior frontal cortices in association with *MAPT* mutations and involvement of a distributed thalamo-cerebello-cortical network in association with *C9orf72* mutations.[4,46] The more severe naming and semantic deficits in the *MAPT* subgroup compared with the *C9orf72* subgroup would be in line with these neuroanatomical signatures and also with previously documented cases of patients with *MAPT* mutations and a clinical phenotype overlapping bvFTD and svPPA.[47]

Taken together, the present findings suggest that involvement of distributed cortical networks mediating verbal semantic processing may underpin the language profile of bvFTD and further suggest that language impairment may be a relevant clinical issue in these patients. Case numbers in this study were relatively small: future work should address language functions prospectively and systematically in larger bvFTD cohorts. Like all work of this kind, the present study was potentially susceptible to patient floor-performance and healthy control ceiling-performance effects associated with conventional neuropsychological tests of language function: this issue will only be fully addressed through development of new graded difficulty tests that can capture the very wide range of performance across target groups in the relevant language domains.

It is worth noting that bvFTD represents a diverse clinicopathological spectrum, and there are inevitably certain limitations in averaging performances at a group level.[48] It will be of particular interest in future work to assess patients with progressive supranuclear palsy and corticobasal degeneration who may exhibit prominent verbal adynamia and speech production deficits[49]; and patients with defined genetic mutations, as such cases potentially illustrate the molecular phenotype of specific brain network disintegration[50].

The potential role of language indices as biomarkers in bvFTD should be further assessed longitudinally over longer periods of follow-up and particularly in genetic mutation carriers at presymptomatic and earliest clinical disease stages. The identification of language deficits in bvFTD complements previous work delineating behavioral features in PPA syndromes[49] and supports the concept of these syndromes as network-based proteinopathies that may transcend conventional syndromic boundaries.[5,50]. Our findings underline the potential for substantial syndromic overlap within the frontotemporal lobar degeneration spectrum, with implications for current diagnostic formulations.

## Supplementary Material

Supplementary Material

## Figures and Tables

**Table 1:** Summary of previous studies assessing language functions in behavioral variant frontotemporal dementia

Study	No. of cases, groups	Methods	Key findings
Cooke et al., 2003 [6]	5 bvFTD, 3 nfvPPA, 11 controls	Functional MRI of sentence processing	bvFTD showed less recruitment of left dorsal inferior frontal cortex than controls
Silveri et al., 2003 [7]	17 bvFTD, 42 AD, 34 controls.	Tests of object and action naming and comprehension	bvFTD impaired on noun and verb naming and comprehension relative to controls
Grossman et al., 2004 [8]	14 bvFTD, 7 nfvPPA, 9 CBS, 8 svPPA, 12 AD, 25 controls	Tests of confrontation naming, lexical retrieval, semantic category judgement; voxel based morphometry	bvFTD impaired on confrontation naming relative to controls. *Neuroanatomy*: naming correlated with grey matter atrophy in left anterior cingulate, left parietal, bifrontal, right temporal regions
McMillan et al., 2004 [9]	14 bvFTD, 8 nfvPPA, 7 svPPA, 25 controls	Confrontation naming task; voxel-based morphometry	bvFTD impaired on naming relative to controls. *Neuroanatomy:* impaired naming correlated with grey matter atrophy in right dorsolateral prefrontal cortex
Grossman et al. 2005 [10]	8 bvFTD, 5 nfvPPA, 3 svPPA	Sentence processing tasks	bvFTD impaired on sentence comprehension, correlated with performance on working memory and executive measures
Cotelli et al., 2006 [11]	16 bvFTD, 2 nfvPPA, 10 CBS, 10 PSP, 6 svPPA, 10 AD, 10 controls	Confrontation naming of pictures	bvFTD impaired on naming, actions more impaired than objects
Blair et al., 2007 [12]	20 bvFTD, 54 nfvPPA, 10 svPPA, 105 AD	Western Aphasia Battery, administered longitudinally	No differences between bvFTD and AD groups at baseline; bvFTD subsequently impaired on spontaneous speech fluency, sentence completion, word recognition
Cotelli et al., 2007 [13]	9 bvFTD, 15 PSP, 11 CBS, 4 svPPA, 10 AD, 10 controls	Tests of violations of semantic coherence, grammatical constructions, general language tests	bvFTD not impaired relative to controls
Murray et al., 2007 [14]	8 bvFTD, 6 nfvPPA, 11 svPPA, 17 controls	Assessment of acquisition of grammatical, semantic and thematic associations to a novel verb, general neuropsychology tests	bvFTD impaired in forming verb associations but not impaired on grammatical sentence judgement or semantic picture-word matching relative to controls
Peelle et al., 2007 [15]	7 bvFTD, 6 nfvPPA, 20 controls	Online word-monitoring paradigm to assess sentence processing	bvFTD showed partial sensitivity to grammatical errors but not thematic violations
Peelle et al. 2008 [16]	32 bvFTD, 28 nfvPPA, 28 svPPA, 29 controls	Sentence comprehension, working memory measures; voxel-based morphometry	Subgroup of bvFTD impaired on comprehension of complex sentences. *Neuroanatomy:* no correlation with sentence comprehension in bvFTD
Garcin et al., 2009 [17]	91 bvFTD	Survival analysis of bvFTD patients with data on clinical features at presentation	Semantic deficits, word-finding difficulty and general language impairment associated with shorter survival in bvFTD
Gunawardena et al., 2010 [18]	12 bvFTD, 16 nfvPPA, 13 controls	Semi-structured speech and neuropsychological measures of executive functioning and language; cortical thickness	bvFTD produced less words per minute (WPM) than controls, and significantly impaired on semantic comprehension but not confrontation naming, relative to controls. *Neuroanatomy:* bvFTD showed more anterior cortical thinning (including significant medial frontal thinning) than nfvPPA. No relationships observed between reduced WPM and cortical thinning
Hughes et al., 2011 [19]	12 bvFTD, 16 controls	General neuropsychology tests; categorical semantic judgements during magnetoencephalography	bvFTD impaired on naming and semantic decisions. *Neuroanatomy:* reduced activity of left frontoparietal network correlated with measures of semantic association
Hsieh et al., 2012 [20]	8 bvFTD, 8 svPPA, 12 AD, 15 controls	Tests of emotion word, abstract and concrete non-emotion word comprehension	bvFTD impaired relative to controls and svPPA on emotion word associations only
Kaiser et al., 2013 [21]	11 bvFTD, 10 AD	Tests of proverb interpretation, other executive and semantic tests; tensor-based morphometry	bvFTD impaired on proverb interpretation relative to AD, correlating with semantic measures. *Neuroanatomy:* impaired proverb interpretation correlated with L > R anterior temporal lobe atrophy
Roca et al., 2013 [22]	35 bvFTD, 14 controls	Tests of executive functions, confrontation naming, semantic knowledge	More severe deficits in ‘low-functioning’ bvFTD subgroup, particularly affecting confrontation naming

Key: AD, Alzheimer’s disease; bvFTD, behavioral variant frontotemporal dementia; CBS, corticobsasal syndrome; nfvPPA, nonfluent variant of primary progressive aphasia; PSP, progressive supranuclear palsy; svPPA, semantic variant of primary progressive aphasian applied) (current diagnostic classifications have been applied).

**Table 2:** Demographic and general cognitive characteristics of participant groups.

Characteristic		Controls	svPPA	nfvPPA	bvFTD
**Demographics**					
No. (male:female)		24 (9:15)	14 (7:7)*	18 (14:4)*	**24 (20:4)**
Handedness (R:L)		13:3	12:2	18:0	20:4
Age (years)		63.8 (7.8)	66.0 (6.7)	68.5 (9.0)	64.6 (7.7)
Education (years)		15.3 (2.9)	13.6 (3.2)	13.8 (3.1)	14.8 (3.8)
Symptom duration (years)		NA	6.7 (4.1)	5.7 (5.2)	7.8 (5.2)
**General cognitive functions**					
MMSE (/30)		30 (0.6)	**21 (6.5)**	**23 (8.7)**	**24 (5.7**)
WASI	Verbal IQ	120 (7)	**71 (18)** *	**73 (13)** *	**83 (22)**
	Performance IQ	116 (9)	**101 (19)**	**92 (17)**	**93 (21)**
Stroop	Color naming (seconds)	30 (4.9)	**52 (24.5)** *	**70 (17.8)** * †	**40 (15.3)**
	Word reading (seconds)	22 (2.8)	**31 (11.5)**	**58 (20.7)** * †	**28 (15)**
	Response suppression (seconds)	57 (12.9)	**98 (46.7)**	**135 (48)** * †	**88 (38.4)**
Episodic memory	RMT Faces (/50)	43 (4.6)	**34 (8)**	**36 (6.3)**	**33 (6.9)**
	RMT Words (/50)	48 (2.3)	**33 (7.3)** ‡	**41 (6.7)**	**35 (7.5)**
Working memory	DS forward	7 (0.9)	6.5 (1.3)	**4.7 (1.3)** * †	**6.3 (1.5)**
	DS reverse	5.1 (0.9)	4.9 (1.5)	**2.5 (1.6)** * †	**4.1 (1.8)**
	SS forward	5.3 (0.9)	**4.6 (1.2)**	**4 (1)** *	5 (1.5)
	SS reverse	5.5 (0.8)	**4.5 (1.2)**	**3.4 (1.5)** * †	**4.3 (1.5)**
Arithmetic	GDA (/24)	15 (4.3)	**8.4 (8.2)**	**4.1 (2.9)** * †	**9.9 (7.1)**

Mean (standard deviation) values are presented and for neuropsychological tests the maximum score is indicated in parentheses, unless otherwise indicated. Significant differences (p<0.05) from healthy control values are in **bold**;

* significantly different (p<0.05) from bvFTD group;

† significantly different (p<0.05) from svPPA group;

‡ significantly different (p<0.05) from nfvPPA group.

bvFTD, behavioral variant frontotemporal dementia; DS, Digit Span; GDA, Graded Difficulty Arithmetic test; MMSE, Mini-Mental State Examination; nfvPPA, nonfluent variant primary progressive aphasia; RMT, Recognition Memory Test; SS, Spatial Span; svPPA, semantic variant primary progressive aphasia; WASI, Wechsler Abbreviated Scale of Intelligence. Details of numbers of participants in each group completing each test are provided in Supplementary Material on-line.

**Table 3:** Language characteristics of participant groups.

Characteristic		Controls	svPPA	nfvPPA	bvFTD
**Language**					
*Pass/ fail tests*					
Auditory input	PALPA3 (≥34/36)^a^	96%	83%	**50%**	77%
Word retrieval	Verb naming (24/24)	100%	**0%** *	**24%** *	**64%**
Repetition	Polysyllabic words (≥38/45)	96%	92%	**18%** * †	86%
	Sentences (10/10)	92%	62%	**20%** * †	83%
Reading	Nonwords (≥23/25)	96%	**64%**	**25%** * †	**64%**
*Graded difficulty tests*					
Comprehension: Single words	BPVS (/150)	147 (2.5)	**72 (50.8)** * ‡	126 (34.1)	123 (33.5)
	Synonyms: Concrete (/25)^a^	24 (1.2)	**13 (6)** * ‡	21 (3.6)	**19 (4.7)**
	Synonyms: Abstract (/25)^a^	24 (1.8)	**14 (5)** * ‡	20 (4.6)	20 (5)
Sentences	PALPA55 (/24)^b^	23 (1.2)	**20 (5.1)** *	19 (4.5)	22 (3.1)
Word retrieval	GNT (/30)	26 (3.5)	**0.3 (0.6)** * ‡	**11 (9.9)**	**11 (8.3)**
Reading	NART/ Schonell (IQ)	122 (4)	**92 (21)** *	**90 (27)** *	**105 (19)**
Spelling	GST (/30)	26 (2.6)	**11 (9.1)** *	**14 (9.6)**	**18 (10)**
**Propositional speech** ^¶^					
	total words	250 (131)	227 (148)	**69 (56)**	183 (118)
	total nouns/total words	0.14 (0.04)	0.13 (0.04)	0.16 (0.07)	0.14 (0.03)
	total verbs/total words	0.16 (0.03)	0.21 (0.03)	0.18 (0.08)	0.18 (0.03)
	mean words/prompt^§^	238 (144)	**75 (56)**	**27 (36)**	**79 (50)**
	median word frequency^¶^	31 (4.5)	**45 (9.8)** *	**46 (4.6)** *	36 (13.6)

Mean (standard deviation) scores are presented and the maximum score is indicated in parentheses, unless otherwise indicated. For variables that healthy control subjects are expected to score at or near ceiling, values represent percentages of people in each group scoring at or above the minimum score anticipated for a healthy control (given in parentheses). Significant differences (p<0.05) from healthy control values are in **bold**;

* significantly different (p<0.05) from bvFTD group;

† significantly different (p<0.05) from svPPA group;

‡ significantly different (p<0.05) from nfvPPA group.

§ total number of words produced / number of prompts from experimenter;

¶ all values ×10^3^;

a chance-level performance 50%;

b chance-level performance 33%;

BPVS, British Picture Vocabulary Scale; bvFTD, behavioral variant frontotemporal dementia; GNT, Graded Naming Test; GST, Graded Difficulty Spelling test; NART, National Adult Reading Test; nfvPPA, nonfluent variant primary progressive aphasia; PALPA, Psycholinguistic Assessment of Language Performance in Aphasia; svPPA, semantic variant primary progressive aphasia. Details of numbers of participants in each group completing each test are provided in Supplementary Material on-line.

**Table 4:** Characteristics of genetic subgroups with behavioral variant frontotemporal dementia

Characteristic		*MAPT*	*C9orf72*
**Demographics**			
No. (male:female)		4:2	3:1
Handedness (R:L)		6:0	4:0
Age (years)		61.5 (3.9)*	67.5 (3.1)
Education (years)		16.3 (4.4)	14.8 (4.1)
Symptom duration (years)		8.7 (5.9)	9.8 (7)
**General cognitive**			
MMSE (/30)		**25 (5)**	**25 (4.7)**
WASI	Verbal IQ	**83 (19.7)**	**84 (20.6)**
	Performance IQ	**98 (10.3)**	**88 (27)**
Episodic memory	RMT Faces	**33 (10.4)**	**33 (6.4)**
	RMT Words	**30 (6.7)** *	**39 (2.4)**
Working memory	DS forward	7 (0.9)	**5.3 (0.5)** *
	DS reverse	5 (1.4)	**4 (0.8)**
	SS forward	5.8 (0.8)	**4.3 (1.5)** *
	SS reverse	5 (0.9)	**4.3 (1.5)**
Arithmetic	GDA (/24)	**11 (5.1)**	**9.8 (8.3)**
**Language skills**			
*Pass/fail variables*			
Auditory input	PALPA3 (≥34/36)	83%	100%
Repetition	Polysyllabic words (≥38/45)	100%	100%
Word retrieval	Verb naming (20/20)	**67%**	**75%**
Reading	Nonwords (≥23/25)	**50%**	75%
*Graded difficulty tests*			
Comprehension: Single words	BPVS (/150)	**120 (24)** *	141 (6.6)
	Synonyms: Concrete (/25)	**20 (5.5)**	22 (2.1)
	Synonyms: Abstract (/25)	**20 (4.5)**	22 (2.1)
Sentences	PALPA55 (/24)	23 (0.5)	22 (2.4)
Word retrieval	GNT (/30)	**5 (6)** *	20 (4.6)
Reading	NART/Schonell (IQ)	**105 (19.7)**	**111 (8.7)**
Spelling	GST (/30)	**14 (10.9)**	24 (0)

Data are presented for patient subgroups with defined mutations in the *MAPT* or *C9orf72* genes. Mean (standard deviation) values and raw scores for neuropsychological tests are presented unless otherwise indicated; maximum scores are indicated in parentheses. For variables that healthy control subjects are expected to score at or near ceiling, values represent percentages of people in each group scoring at or above at or above the minimum score anticipated for a healthy control (given in parentheses). Significant differences (p<0.05) for each generic subgroup compared with healthy control values are in **bold**;

* significantly worse than other genetic subgroup (p<0.05).

BPVS, British Picture Vocabulary Scale; DS, Digit Span; GDA, Graded Difficulty Arithmetic test; GNT, Graded Naming Test; GST, Graded Difficulty Spelling test; MMSE, Mini-Mental State Examination; NART, National Adult Reading Test; PALPA, Psycholinguistic Assessment of Language Performance in Aphasia; RMT, Recognition Memory Test; SS, Spatial Span; WASI, Wechsler Abbreviated Scale of Intelligence.

**Table 5:** Longitudinal comparisons of language performance in participant groups

Characteristic	Time-point	Controls	svPPA	nfvPPA	bvFTD
Inter-test interval (days)	-	471 (190)	518 (236)	409 (110)	570 (212)
BPVS (/150)	1	148 (1.2)	**87 (47.8)**	143 (4.9	128 (28.6)
	2	147 (2.2)	**67 (50.1)**	131 (27.9)	132 (16.2)
Concrete synonyms (/25)	1	24 (0.7)	14 (7.6)	21 (1.8)	21 (2.8)
	2	24 (0.8)	15 (3.7)	21 (2.6)	21 (4.2)
Abstract synonyms (/25)	1	24 (0.9)	17 (5.8)	20 (3.7)	22 (4.7)
	2	24 (0.8)	15 (3.7)	19 (4.8)	22 (2.4)
PALPA 55 (/24)	1	24 (0.8)	22 (3.4)	**21 (2.5)**	22 (1.9)
	2	24 (0.7)	22 (2.3)	**17 (6.3)**	22 (1.5)
GNT (/30)	1	26 (3.6)	0.4 (0.7)	18 (6.6)	**13 (8.4)**
	2	26 (3.6)	0 (0)	12 (10.1)	**10 (8.3)**

Values denote mean (standard deviation) raw scores for neuropsychological tests unless otherwise indicated; maximum scores are indicated in parentheses. Significant differences (p<0.05) between time-points within patient groups are indicated in **bold**. Details of numbers of participants in each group completing each test are provided in Supplementary Material on-line. BPVS, British Picture Vocabulary Scale; GNT, Graded Naming Test; PALPA, Psycholinguistic Assessment of Language Performance in Aphasia.

**Table 6:** Neuroanatomical associations of language deficits in patient groups

Language test	Brain region	Side	Cluster size (voxels)	Peak (mm)			Z score	P value
				x	y	z		
**bvFTD**								
GNT	Superior parietal lobe	R	162	27	−60	46	5.22*	0.01
	Anterior fusiform gyrus	L	5662	−34	−12	−42	5.13*	0.014
Synonyms	Inferior frontal gyrus / sulcus	L	146	−44	46	9	4.43	0.011
**nfvPPA**								
GNT	Middle temporal gyrus/superior temporal sulcus	L	829	−50	−56	8	4.62	0.005

The table shows grey matter associations identified in the voxel-based morphometric analysis of language test performance in the patient cohort. All clusters greater than 100 voxels in size are shown with coordinates of local maxima in MNI space;

* significant at p<0.05_FWE_ after multiple comparisons over whole brain; other associations significant at p<0.05_FWE_ after multiple corrections within the pre-specified region of interest (see text).

bvFTD, behavioral variant frontotemporal dementia; GNT, Graded Naming Test (nouns); nfvPPA, nonfluent variant primary progressive aphasia; Synonyms, test of single word comprehension (see text and Supplementary Materials for details).
