# Supplementary material for: The language profile of behavioral variant frontotemporal dementia
Source: J Alzheimers Dis. Author manuscript; Available in PMC 2016 Feb 4. (PMC4740928; doi:10.3233/JAD-150806)
Supplement: Supplementary Material [file NIHMS66619-supplement-01.pdf]

SUPPLEMENTARY MATERIAL
The language profile of behavioral variant frontotemporal dementia, by CJ Hardy et al

Details of language test procedures

Speech input processing was assessed using the Psycholinguistic Assessments of Language Processing in Aphasia Test 3 (PALPA3)[24] word minimal pairs discrimination test (36 trials): this test of phoneme perception required participants to decide whether pairs of spoken monosyllabic words are the same (e.g. 'cat' – 'cat') or different (e.g. 'cat' – 'tack').

Speech repetition was assessed using the polysyllabic word repetition task[25] (45 trials) comprising single words of one, two and three syllables in length. The subject was required to repeat the word (e.g. skeleton) back to the examiner verbatim.

Single word comprehension was assessed using concrete nouns and abstract words from the synonyms comprehension test[27] (25 trials in each noun category): on each trial participants were required to judge which of two response words (presented both spoken and written) most closely matched the probe word (e.g. for concrete, chapel: altar/ church? for abstract, macabre: ugly/ gruesome?)

Sentence comprehension was assessed using the PALPA55[24] picture-sentence matching task (24 trials): on each trial participants were shown three pictures of a scenario and required to identify which picture matched a verbal sentence description.

Noun naming was assessed using the Graded Naming Test (GNT)[28] (30 trials): in this picture naming task items of graded difficulty were presented, ranging from highly familiar (e.g. 'kangaroo') to relatively unfamiliar (e.g. 'sextant').

Reading was assessed using the Graded Nonword Reading test[31] (25 trials): participants were presented with nonwords of graded difficulty from one syllable to three syllables in length. Word

reading was assessed with the National Adult Reading Test (NART)[18]. The Schonell Graded Word Reading Test[19] was also administered if participants scored <15 on the NART.

Spelling was assessed using the Graded Difficulty Spelling test[32] (30 trials): participants were presented with trials of graded difficulty each comprising the target word, followed by its use in a sentence, followed by the target word again (e.g., "Said. I said I would come. Said.").

Verb naming was assessed using a task developed in-house, in which participants were shown picture of actions (e.g., a man running) and asked to name the verb (20 trials; see example below). Healthy subjects are expected to perform at ceiling on this task.

Task instruction: "I have a set of pictures here, in each one I would like you to tell me what the person is doing. They are all –ing words".

E.g. "Smelling"

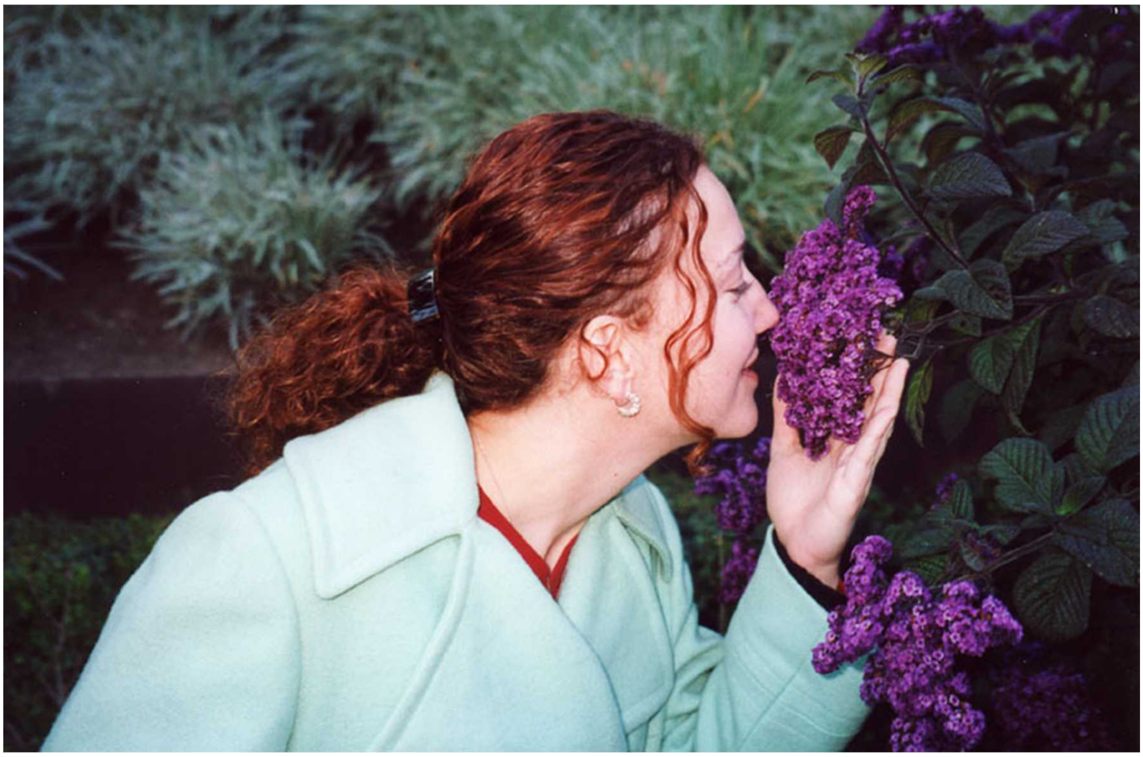

Table S1. Data for subgroups of more and less severely affected bvFTD patients

**Characteristic**	**More severe**	**Less severe**
No.	10	14
Age (years)	**60 (4.6)**	**68 (8.2)**
Symptom duration (years)	**9 (4.6)**	**6.9 (5.6)**
Brain MRI atrophy profile		
Focal left temporal	1	0
Focal right temporal	0	1
Bilateral temporal	3	5
Bilateral frontal	4	3
Right > left frontotemporal	0	3
Bilateral frontotemporal	2	2
MMSE (/30)	**19.7 (5.3)**	**27.4 (3.2)**
GNT (/30)	**6.3 (6.8)**	**14.7 (7.5)**
Time-point 1	7.3 (8)	**14.7 (8)**
Time-point 2	7.8 (9)[a]	**11.3 (8)***[b]
Synonyms: Concrete (/25)	**14.3 (3.7)**	**22.4 (1.3)**

'More severe' is here defined as those patients performing >2 standard deviations below the healthy control group mean on tests of naming and single word comprehension. Mean (standard deviation) values are presented unless otherwise indicated. [a]n=4; [b]n=12. GNT, Graded Naming Test; MMSE, Mini-Mental State Examination Score. Significant differences between subgroups are indicated in bold; *significant difference between time-points.

Participant numbers

Numbers of participants in each group completing each of the tests listed in Tables 2 to 5 were as follows:

Table 2

Healthy controls: MMSE (16), GST (23), GDA (23), SS forward (23), SS reverse (23) Handedness information also only available for a subset (16).

svPPA: Stroop color naming (12), Stroop word reading (13), Stroop response suppression (12), RMT Faces (13), RMT Words (13).

nfvPPA: Stroop color naming (12), Stroop word reading (13), Stroop response suppression (10), RMT faces (17), DS forward, DS reverse, SS forward, SS reverse (all 17), NART/ Schonell (16), nonword reading (16), GST (16), GDA (14).

bvFTD: Stroop color naming (22), Stroop word reading (22), Stroop response suppression (20), RMT faces (22), RMT words (21), SS forward (21), SS reverse (21), nonword reading (22), GST (22).

Table 3

Healthy controls: Propositional speech (15).

svPPA: PALPA3 (12), word repetition (13), Synonyms concrete (12), Synonyms abstract (12), Propositional speech (4)

nfvPPA: PALPA3 (16), Verb naming (16), word repetition (17), sentence repetition (13), Synonyms concrete (17), Synonyms abstract (22), PALPA55 (22), Propositional speech (7).

bvFTD: PALPA3 (22), word repetition (21), sentence repetition (23), Synonyms concrete (23), Synonyms abstract (22), PALPA55 (21), Propositional speech (9).

Table 4

MAPT: RMT faces (5), RMT words (5), Synonyms concrete (5), Synonyms abstract (5), PALPA55 (5).

C9orf72: SS forward (3), SS reverse (3), GST (3), PALPA 3 (3), Word repetition (2).

Table 5

Healthy controls: GNT (15), Synonyms concrete (12), Synonyms abstract (13), PALPA 55 (12), BPVS (13).

svPPA: GNT (9); Synonyms concrete (5); Synonyms abstract (5); PALPA 55 (6); BPVS (9).

nfvPPA: GNT (8); Synonyms concrete (10); Synonyms abstract (10); PALPA 55 (9); BPVS (10).

bvFTD: GNT (16); Synonyms concrete (11); Synonyms abstract (12); PALPA 55 (8); BPVS (16).

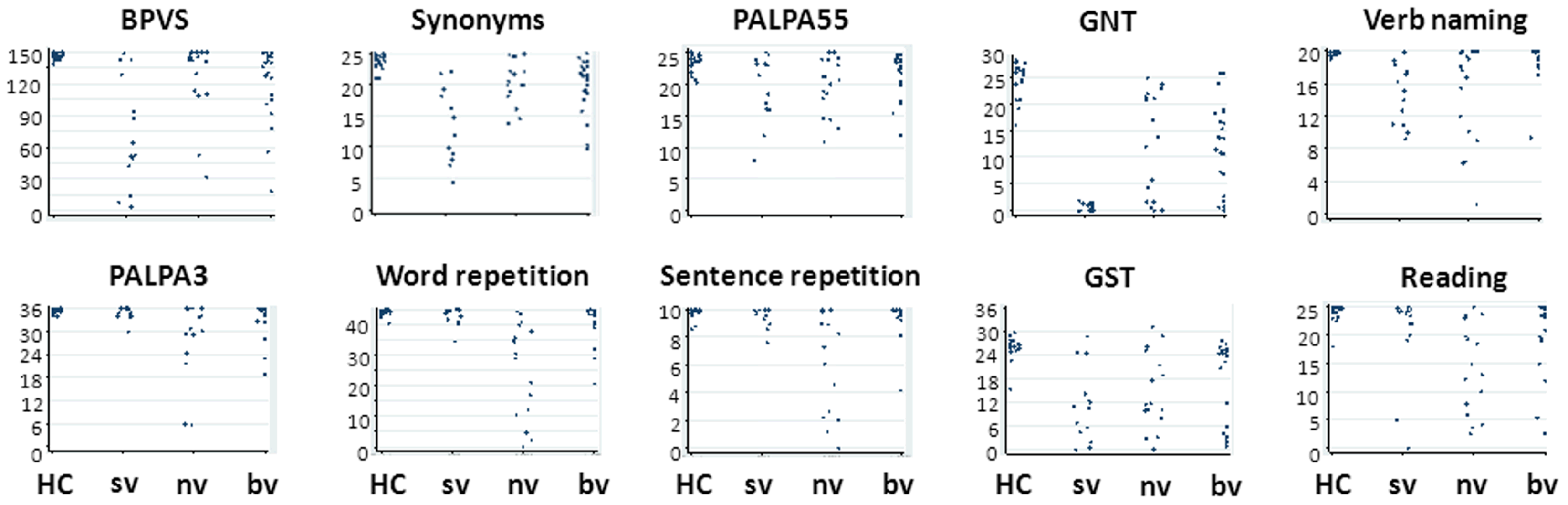

Figure S1. Individual raw performance data on language tests are plotted for each participant group; y-axes show test scores (note change of scale between tests). BPVS, British Picture Vocabulary Scale; bv, behavioral variant frontotemporal dementia; GNT, Graded Naming Test; GST, Graded Spelling Test; HC, healthy controls; nv, nonfluent variant of primary progressive aphasia; PALPA, Psycholinguistic Assessment of Language Processing in Aphasia (PALPA3, minimal pairs discrimination; PALPA55, sentence comprehension); Reading, nonword reading; sv, semantic variant of primary primary progressive aphasia; Synonyms, concrete noun comprehension (data for abstract nouns not shown). See text for further details.
